# Killing of Trypanosomatid Parasites by a Modified Bovine Host Defense Peptide, BMAP-18

**DOI:** 10.1371/journal.pntd.0000373

**Published:** 2009-02-03

**Authors:** Lee R. Haines, Jamie M. Thomas, Angela M. Jackson, Brett A. Eyford, Morteza Razavi, Cristalle N. Watson, Brent Gowen, Robert E. W. Hancock, Terry W. Pearson

**Affiliations:** 1 Department of Biochemistry and Microbiology, University of Victoria, Victoria, British Columbia, Canada; 2 Department of Biology, University of Victoria, Victoria, British Columbia, Canada; 3 Department of Microbiology and Immunology, University of British Columbia, Vancouver, British Columbia, Canada; National Institute of Allergy and Infectious Diseases, United States of America

## Abstract

**Background:**

Tropical diseases caused by parasites continue to cause socioeconomic devastation that reverberates worldwide. There is a growing need for new control measures for many of these diseases due to increasing drug resistance exhibited by the parasites and problems with drug toxicity. One new approach is to apply host defense peptides (HDP; formerly called antimicrobial peptides) to disease control, either to treat infected hosts, or to prevent disease transmission by interfering with parasites in their insect vectors. A potent anti-parasite effector is bovine myeloid antimicrobial peptide-27 (BMAP-27), a member of the cathelicidin family. Although BMAP-27 is a potent inhibitor of microbial growth, at higher concentrations it also exhibits cytotoxicity to mammalian cells. We tested the anti-parasite activity of BMAP-18, a truncated peptide that lacks the hydrophobic C-terminal sequence of the BMAP-27 parent molecule, an alteration that confers reduced toxicity to mammalian cells.

**Methodology/Principal Findings:**

BMAP-18 showed strong growth inhibitory activity against several species and life cycle stages of African trypanosomes, fish trypanosomes and *Leishmania* parasites *in vitro*. When compared to native BMAP-27, the truncated BMAP-18 peptide showed reduced cytotoxicity on a wide variety of mammalian and insect cells and on *Sodalis glossindius*, a bacterial symbiont of the tsetse vector. The fluorescent stain rhodamine 123 was used in immunofluorescence microscopy and flow cytometry experiments to show that BMAP-18 at low concentrations rapidly disrupted mitochondrial potential without obvious alteration of parasite plasma membranes, thus inducing death by apoptosis. Scanning electron microscopy revealed that higher concentrations of BMAP-18 induced membrane lesions in the parasites as early as 15 minutes after exposure, thus killing them by necrosis. In addition to direct killing of parasites, BMAP-18 was shown to inhibit LPS-induced secretion of tumour necrosis factor alpha (TNF-α), a cytokine that is associated with inflammation and cachexia (wasting) in sleeping sickness patients. As a prelude to *in vivo* applications, high affinity antibodies to BMAP-18 were produced in rabbits and used in immuno-mass spectrometry assays to detect the intact peptide in human blood and plasma.

**Conclusions/Significance:**

BMAP-18, a truncated form of the potent antimicrobial BMAP-27, showed low toxicity to mammalian cells, insect cells and the tsetse bacterial symbiont *Sodalis glossinidius* while retaining an ability to kill a variety of species and life cycle stages of pathogenic kinetoplastid parasites *in vitro*. BMAP-18 also inhibited secretion of TNF-α, an inflammatory cytokine that plays a role in the cachexia associated with African sleeping sickness. These findings support the idea that BMAP-18 should be explored as a candidate for therapy of economically important trypanosome-infected hosts, such as cattle, fish and humans, and for paratransgenic expression in *Sodalis glossinidius*, a bacterial symbiont in the tsetse vector, as a strategy for interference with trypanosome transmission.

## Author Summary

Protozoan parasites cause serious diseases in large areas of the tropics. Control of these diseases depends to a great extent on the use of therapeutic drugs, many of which are highly toxic. In addition, parasite resistance to several of the front-line drugs is increasing. Host defense peptides (HDP; formerly called antimicrobial peptides) have recently received attention as potential anti-parasite effector molecules. We earlier reported that one such peptide, bovine myeloid antimicrobial peptide (BMAP-27), is a potent inhibitor of the growth of trypanosomes and *Leishmania in vitro*. Here we report our studies on BMAP-18, a truncated form of BMAP-27, which showed reduced toxicity to mammalian and insect cells and yet retained its direct toxicity to parasites *in vitro*. BMAP-18 also strongly inhibited LPS-induced release of tumour-necrosis factor alpha (TNF-α) from human leukocytes, and thus has immunomodulatory activity. These findings suggest that BMAP-18 has potential as a therapeutic agent for treatment of infected animals or as an inhibitor of parasite transmission by their insect vectors. In anticipation of using BMAP-18 *in vivo*, we have also developed high affinity antibodies to BMAP-18 and have shown that these can be used, in conjunction with mass spectrometry, to detect the peptide in whole blood or plasma.

## Introduction

Trypanosomatid protozoan parasites cause a variety of diseases that affect the livelihood of people in vast areas of the world. Control of such diseases depends, to a large extent, on a small set of prophylactic and therapeutic anti-parasite drugs [1]. However, as with many microbes, inappropriate use of these agents has led to an alarming increase in parasite resistance, for example with African trypanosomes and *Leishmania* [2],[3]. This has created a need for novel compounds to prevent and cure diseases caused by these predominantly tropical parasites. An ideal anti-parasite agent would have a broad spectrum of activity, would be largely unaffected by mutations in the target microbe and be non-toxic *in vivo*. Effectors with such potential are the host defense peptides (HDP), ancient and highly successful molecules that remain functional over long periods of evolution, suggesting that microbial resistance to them is not inevitable.

HDP are widely distributed elements of immunity that show a broad spectrum of activity against a variety of bacteria, fungi, parasites, enveloped viruses and transformed cells [4]. HDP are evolutionarily conserved peptides that have received much attention from the medical research community for their versatility and potential as alternatives to existing antimicrobials, many of which have become less effective due to increasing resistance in the target populations [2],[3]. In some cases, HDP have advantages over traditional drugs and antibiotics, including having a much wider spectrum of activity and an apparent robustness against resistance. Most importantly, HDP naturally occupy positions in immunity as innate immune mediators [4] and thus may show pleiotropic activity in controlling infections.

HDP are expressed by a variety of cells as holoproteins that are cleaved proteolytically to release the active peptides. An impressive repertoire of more than 1000 HDP have been reported in the antimicrobial peptide database [5],[6]. Many of these molecules target pathogens directly by disturbing the pathogen's membrane potential or by interfering with cell functioning internally, thus causing cell death by either necrosis or apoptosis. Perhaps as important, because many HDP selectively modulate host innate immune responses, they may exhibit multi-pronged effector functions. HDP, many of which were first detected by their direct antimicrobial properties, show variable chemotactic influences on a variety of immune cells including monocytes, neutrophils, eosinophils and T cells [7],[8]. In addition, HDP can be potent and selective anti-inflammatory agents, that suppress the production of pro-inflammatory cytokines in response to lipopolysaccharide, and can enhance protective immunity and promote the transition to adaptive immunity [4]. The local antimicrobial protection conferred by peptides released at the site of challenge is enhanced by the complex interaction of these peptides with innate and adaptive elements of the immune system [8].

The cathelicidins are a family of HDP, some of which are produced and stored in the neutrophils of many mammalian species [9]. All members of the cathelicidin family contain a C-terminal cationic domain and an N-terminal cathelin portion that must be cleaved to release the active C-terminal peptide. Bovine myeloid antimicrobial peptide-27 (BMAP-27) [10] is one such active peptide (not to be confused with the very different BMAP-28 molecule [10]), with a predicted cationic N-terminal amphipathic helix and a hydrophobic C-terminal tail. BMAP-27 has been shown to have potent antimicrobial activity on bacteria [10], fungi [11], chlamydia [12] viruses [13] and parasites [14],[15]. However, BMAP-27 showed some cytotoxic activity on human cells (erythrocytes and neutrophils [10]) and thus was subsequently modified by cleaving off the C-terminal hydrophobic tail to reveal a potent antimicrobial peptide, BMAP-27 [1]–[18] (hereafter called BMAP-18), which demonstrated reduced toxicity to human cells.

Previous work in our lab showed that African trypanosomes were effectively killed *in vitro* with low concentrations of the BMAP-27 peptide, although cytotoxicity to mammalian cells was also observed [15]. The strong anti-parasite activity observed with BMAP-27 inspired us to further investigate BMAP-27 and its truncated form, BMAP-18, for their effects on a variety of species and life cycle stages of kinetoplastid parasites and on a range of mammalian and insect cell lines. Our goal was to qualify BMAP-18 as an effective *in vitro* anti-parasite effector and to lay the groundwork for further investigations into using BMAP-18 as a novel therapeutic *in vivo*. Our results support the idea that BMAP-18 is a strong candidate for possible use as a therapeutic in animals for treating trypanosome infections or in the tsetse vector as a parasite transmission blocking agent.

## Materials and Methods

### Trypanosomes

Bloodstream forms (BSF) of *T. b. brucei* 427.01 [16] were obtained from Dr. Sam Black (Amherst, MA) and adapted to growth *in vitro*. In brief, upon thaw, BSF trypanosomes (a dilution series from 10^6^–10^3^/mL) were grown at 37°C in flat-bottomed 24-well tissue culture plates (Falcon 3047, Becton-Dickinson, Lincoln Park, NJ) in modified Baltz's medium supplemented with 10% heat-inactivated fetal bovine serum (FBS) [17]. Trypanosomes were examined daily in each well and only those wells showing healthy organisms in log-phase of growth were chosen for further subculture and use in minimal inhibitory concentration (MIC) assays (see below). Procyclic culture forms (PCF) of *T. b. brucei* 427.01, *T. b. gambiense* U2 [18], *T. b. rhodesiense* ViTat 1.1 [19], and *T. congolense* IL3000 [20] were derived from their corresponding BSF by transformation at 27°C [21]. Tsetse transmissible *T. b. brucei* TSW 196 PCF [22] were obtained from the Liverpool School of Tropical Medicine. All PCF were cultured in MEM/10% FBS as described [20],[23]. The fish pathogen *T. danilewskyi* strain TrCa was obtained from Mike Belosevic and Debbie Plouffe (University of Alberta) and were grown *in vitro* as described [24].

### Leishmania

Promastigotes of *Leishmania donovani* LD3 [25] were obtained from Dr. Sam Turco (Lexington, KY). *Leishmania* promastigotes were adapted to a modified minimal essential medium (MEM) containing 10% FBS [20],[23] and maintained in culture *in vitro* at 27°C.

### Mammalian cell lines

B lymphocyte hybridomas 2A11 (anti-hypoderma C) and 10B1 (anti-trypanosome tubulin) were of murine origin (BALB/c) and secrete IgG1 mAbs (Pearson lab, unpublished). EL4 thymoma cells were also of murine origin [26]. NIH 3T3 cells were derived from an embryonic fibroblast cell line originally isolated from Swiss mice [27]. NR8383 alveolar cells were derived from a Sprague-Dawley rat lung [28]. HeLa cells were originally established from a cervical carcinoma of human origin [29]. All mammalian cell lines were grown in Dulbecco's Modified Eagles Medium (Gibco BRL cat no. 23700-057) supplemented with 10% FBS, L-glutamine, sodium pyruvate, 5×10^−5^ M 2-mercaptoethanol and penicillin/streptomycin.

### Insect cell lines

CF70 cells were derived from ovarian cultures of the eastern spruce budworm *Choristoneura fumiferana* [30]. Sf9 cells were derived from pupal ovarian tissue of the fall armyworm *Spodoptera frugipera* [31]. FPMI-NL-18 cells were established from the embryonic tissues of the red-headed pine sawfly, *Neodiprion lecontei* [32]. Stocks of all insect cells were obtained from Beatrixe Whittome [Dept. of Biology, UVic]. Insect cell lines were grown in Grace's insect medium [33] containing 15% FBS and 0.25% (w/v) bacto tryptose broth (Difco; Becton Dickinson, Oakville, ON).

### Sodalis glossinidius

*Sodalis glossinidius*, a secondary symbiont of tsetse flies (*Glossina*) [34], were isolated at the University of Alberta according to the protocol of Welburn and Maudlin [35], modified by changing the method used to maintain asepsis prior to initial culture [36]. In brief, *S. glossinidius* from tsetse hemolymph were initially grown on monolayers of *Aedes albopictus* cells and after 20 days in culture were adapted to axenic growth in serum-free medium [36]. The relatively slow growing bacteria were maintained by passaging every 5 days. Clones were isolated by spreading 2 ml of culture aseptically onto Mitsuhashi and Maramorosch (MMI) agar plates (1% (w/v) agar (Difco, Detroit, MI) followed by a 15 min incubation at room temperature to allow the culture to absorb into the agar. The plates were then placed in an unvented BBL GasPak™ anaerobic jar with a BBL CampyPak *Plus™* system (Becton Dickinson, Sparks, MD) to generate microaerophilic conditions. Incubation at 27°C for 7 days resulted in small, white, papillated colonies. Culture purity was monitored by Coomassie Brilliant Blue staining of SDS-PAGE-separated proteins that yield a unique banding pattern [36] and by polymerase chain reaction using *S. glossinidius*-specific primer sets [34].

### BMAP-27 and BMAP-18 host defense peptides

**Figure 1:**
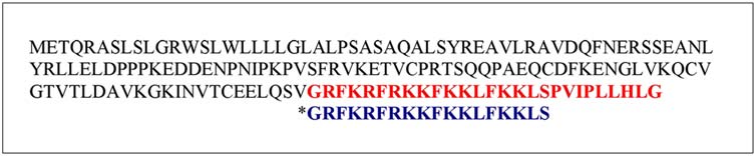
The Amino Acid Sequences of BMAP-27 and BMAP-18 peptides. The entire prepro sequence of the Bovine Myeloid Antimicrobial Peptide-27 (BMAP-27) precursor is shown. The Antimicrobial Peptide Database [6] ID number for BMAP-27 is: AP00366. The N-terminal cathelin domain is proteolytically cleaved to release the C-terminal antimicrobial peptide BMAP-27, shown in red type. The truncated BMAP-18 sequence (designated with the asterisk*, with the C-terminal hydrophobic 9-mer removed) is shown in blue type below the BMAP-27 sequence.

The cathelicidins BMAP-27 and BMAP-27 [1]–[18] (BMAP-18) were synthesized by Fmoc (N-(9-fluorenyl) methoxycarbonyl) chemistry at the University of British Columbia's Nucleic Acid/Protein Services Facility. A comparison of the BMAP-27 and BMAP-18 sequences is shown in Figure 1. BMAP-18 was also synthesized with a C-terminal cysteine for coupling of the peptide to the protein carrier keyhole limpet hemocyanin (KLH) for polyclonal antibody production and for affinity purification of the resultant antibodies (see below). The peptides were analysed for purity by high-performance liquid chromatography and for identity (and purity) by matrix-assisted laser desorption ionization time of flight (MALDI-TOF) mass spectrometry. All peptides were of greater than 80% purity. Lyophilized peptides were dissolved in sterile distilled water to a final concentration of 2.0 mg/mL and dilutions were prepared for inhibition assays, for enzyme-linked immunosorbent assays (ELISA), for affinity purification of antibodies and for immuno-mass spectrometry assays as described below.

### Inhibition of eukaryotic and prokaryotic cells by BMAP-27 and BMAP-18

Minimal Inhibitory Concentration (MIC) assays were performed on trypanosomes, *Leishmania*, mammalian cell lines, insect cell lines and the bacterium *Sodalis glossinidius* using the chromogenic/fluorogenic substrate alamarBlue [37] as previously described [15] with minor changes. In brief, healthy, log phase, BSF and PCF trypanosomes, *Leishmania* promastigotes, mammalian cells and insect cells were adjusted to 2.0×10^4^ cells/mL in fresh medium and 100 µL (2.0×10^3^ cell/well) were dispensed into wells of round-bottom 96-well polypropylene microtitre plates (COSTAR/ Corning Inc, Corning, New York: Cat. No. 3790) containing serial dilutions (10 µL in 0.2% FBS) of the BMAP-27 and BMAP-18 peptides. One exception was that *T. danilewskyi* BSF were adjusted to 5×10^4^ cells/mL and 100 µL (5×10^3^) were seeded per well. After 66 hours of incubation at 37°C (for BSF and mammalian cells) or 27°C (for PCF, *T. danilewskyi*, *Leishmania* promastigotes, insect cells and *S. glossinidius*), 10 µL of alamarBlue (BioSource International, Inc., Camarillo, CA) were added to each well and the plates were incubated for another 6 h (final incubation of 72 h). Seventy µL of cell free supernatant from each well were then transferred into 96-well flat-bottomed black/white microplates (Greiner Bio-One, CellStar, Cat no. 655079, MJS Biolynx, Brockville ON) for fluorescence measurement. Fluorescence was measured using a Cytofluor 2300 microplate reader (Millipore, Bedford, MA) set to an excitation wavelength of 540 nm and an emission wavelength of 590 nm. The alamarBlue assay measures cell viability and proliferation based on detection of metabolic activity [38]. The alamarBlue colorimeteric/fluorometric growth indicator incorporates an oxidation-reduction indicator that changes colour in response to chemical reduction by metabolically active cells. Growth related reduction causes the indicator to change from oxidized (blue, non-fluorescent) to reduced (red, fluorescent).

*S. glossinidius* were cultured in Mitsuhashi and Maramorosch broth (Sigma-Aldrich Canada, Oakville, ON) for 3 days at 27°C in an atmosphere of 5% CO_2_ in air [36]. Cultures were diluted in fresh medium to 5×10^5^ bacteria per mL using the following conversion: OD_600_ of 0.8 = 1×10^9^ bacteria/ml. Diluted bacterial suspensions (100 µL) and peptides (10 µL of each dilution) were loaded into the wells of round-bottom microtitre plates as described above. Because of the slower growth rate of *S. glossidinius*, alamarBlue was added after 72 hours and 6 hours later, the plate contents were transferred into flat-bottomed black/white microplates for fluorescence determination as described above.

### Scanning electron microscopy

Suspensions of parasites (10^6^ in 500 µL PBS containing 25 µg BMAP-18) were incubated for 15 min at 37°C (BSF trypanosomes) or 27°C (PCF trypanosomes or *Leishmania* promastigotes). The treated cell suspensions were mixed with an equal volume of fixative containing 3% formaldehyde and 3% glutaraldehyde in 0.1 M sodium cacodylate buffer and placed in a fridge at 4°C for overnight fixation. The next day, after warming to room temperature, the fixed parasites were centrifuged in 1.5 ml Eppendorf microcentrifuge tubes, the supernatant decanted and replaced with new fixative. This procedure was repeated, to ensure the complete removal of serum from the parasites thus allowing them to bind to polylysine coated slides. An aliquot of each sample was placed onto separate 0.1% poly-l-lysine (Sigma Chemical Company, St. Louis, MO) treated 10 mm circular glass cover slips and these were incubated in a humid chamber for 1 hour. After washing in Karnovsky's cacodylate buffer, the samples were dehydrated in a graded ethanol series and critical point dried using liquid carbon dioxide. The cover slips were glued with silver paste onto labeled aluminum scanning electron microscopy stubs and sputter coated with gold in an Edwards S150B Sputter Coater. Digital images were collected using a Hitachi S-3500N Scanning Electron Microscope operating at 10 kV at 5000× magnification.

### Measurement of mitochondrial activity using fluorescent rhodamine 123

Trypanosomal mitochondrial activity was measured using the fluorescent, cell-permeant, cationic fluorescent dye rhodamine 123 (R-302; Molecular Probes, Eugene, OR) that is sequestered by active mitochondria [39]. Wild type *T. b. brucei* 427.01 PCF (1×10^7^/mL) were incubated at 27°C for 10 minutes with 250 nM (final concentration) rhodamine 123 (Cat. No. R8004, Sigma St Louis, MO) and then BMAP-18 peptide (5 µL of 1.0 mg/mL = 5 µg) was added. At intervals, trypanosomes were analyzed by immunofluorescence microscopy and by flow cytometry. Trypanosomes were first examined by light- and fluorescence microscopy (for observation of rhodamine 123 fluorescence) using a Zeiss Standard binocular microscope fitted with an epifluorescence attachment and a Zeiss NeoFluor 63/1.25 oil immersion objective. Digital photographs were taken with a Nikon CoolPix 2700 camera at high resolution. The images were stored and manipulated in TIFF format using PhotoshopTM 5.5 graphic software (Adobe Systems Inc., San Jose, CA). The same populations of trypanosomes were analyzed for forward scatter (size), side scatter (granularity) and fluorescence (rhodamine 123) using a FACSCalibur flow cytometer (Becton-Dickinson, San Jose, CA). For each sample, a minimum of 10,000 events were analyzed.

### Immunomodulatory effects of BMAP-27 and BMAP-18

Human venous blood (100 mL) was collected from healthy volunteers in Vacutainer collection tubes containing heparin (Cat No. 362753, BD, Franklin Lakes, NJ) according to the University of British Columbia Clinical Research Ethics Board guidelines and approval. The blood was mixed at a 1∶1 ratio with RPMI 1640 medium (supplemented with 10% (v/v) FBS, 2 mM L-glutamine, and 1 mM sodium pyruvate) and the peripheral blood mononuclear cells (PBMC) were separated by centrifugation in Ficoll-Paque™ PLUS (Cat No. 17-1440-02, GE Healthcare). PBMCs were isolated from the buffy coat, washed twice in sterile PBS and the numbers of live cells were determined by trypan blue exclusion. PBMCs (0.1 mL at 1×10^6^ cells/mL) were seeded into 96-well tissue culture dishes (Sarstedt), incubated at 37°C in 5% CO_2_ in air and rested for 2 h before experimental treatment.

To measure cytokine stimulation, PBMCs were treated for 24 h with doubling dilutions (100 µg/mL-1.56 µg/mL) of BMAP-18 and BMAP-27. Monocyte chemotactic protein-1 (MCP-1), the chemotactic cytokine Gro-alpha (Gro-∝), and tumour necrosis factor alpha (TNF-a) were measured in culture supernatants using ELISAs (see below). To test for anti-endotoxin activity, PBMCs were pretreated with 5 µg/mL BMAP-18 or BMAP-27 for 30 minutes prior to the addition of 10 ng/mL of purified *Pseudomonas aeruginosa* LPS for 4 hours. *Pseudomonas aeruginosa* LPS was purified as described previously [40]. After 4 hours, secreted TNF-∝ was measured by ELISA (see below).

All tissue culture supernatants were centrifuged at 1000× g for 10 min to remove cells, aliquoted and then stored at −20°C before assaying for cytokines by ELISA, according to the manufacturer's instructions (MCP-1 and TNF-α, eBioscience Inc., San Diego, CA; Gro-α, R&D Systems, Minneapolis, MN).

### BMAP-18-specific antibodies

Immunization of rabbits was performed to obtain high affinity polyclonal antibodies specific for BMAP-18. Two female New Zealand White rabbits were bled to obtain preimmunization sera and after one day of rest were immunized with BMAP-18-KLH conjugate (subcutaneous injections of 0.25 mg in complete Freund's adjuvant). Four subsequent subcutaneous injections containing 0.25 mg of peptide-KLH conjugate in incomplete Freund's adjuvant were given using standard procedures. At intervals, to determine if an adequate immune response had been achieved, test bleed sera were prepared and titrated using the unconjugated, “immunizing peptides” in an ELISA where the peptide antigens are dried onto assay plates (see “peptide ELISA”, below). Once adequate antibody responses were achieved, antisera were prepared from several bleeds from each rabbit. Affinity purified anti-BMAP antibodies were obtained by elution from an immunoadsorbent made by covalent coupling of BMAP-18 peptide through an added C-terminal cysteine to SulfoLink® Coupling Gel (Cat No. 20401; Pierce, Rockford IL). The antibodies were used in ELISA and in an immuno-mass spectrometry assay (see below) to detect BMAP-18 peptide in human blood or in plasma.

### Peptide ELISA

A modified ELISA was used to measure specific anti-BMAP-18 antibody titres and to detect BMAP-18 in human serum samples. First, a standard ELISA method [41] was modified to use anti-rabbit alkaline phosphatase second antibodies and free peptide antigens (i.e. not coupled to protein carriers) to coat the polystyrene microtitre ELISA plates. In this specialized “peptide ELISA”, peptide was dissolved in distilled water to 5.0 µg/mL and 100 µL (0.5 µg/well) of this solution were dried onto each well by overnight incubation at 37°C in a dry incubator. Dilutions of the rabbit antisera were then used as a source of primary antibodies to determine the titres. To determine if BMAP-18 could be detected in complex protein mixtures, 0.5 µg amounts of BMAP-18 were spiked into 100 µL of serially diluted human serum and dried onto ELISA plates overnight, prior to adding a 1/400 dilution (predetermined from the titration data) of first antibody, followed by second antibody and substrate.

### Detection of BMAP-18 by immuno-mass spectrometry

An immunoenrichment technique coupled with peptide detection by mass spectrometry was also used to detect BMAP-18 in human blood and plasma. We used immuno-matrix assisted laser desorption ionization (iMALDI) time of flight mass spectrometry to detect BMAP-18 by its characteristic mass. To do this, 1 µg of BMAP-18 was spiked into 50 µL aliquots of freshly prepared undiluted human blood, plasma or serum, 2 µg of affinity purified rabbit antibody were added to each sample and the mixtures were incubated (with shaking) for 18 hours at 4°C to allow antibody-peptide binding. Negative controls consisted of all three samples without added peptide. Another negative control consisted of 50 µL of PBS containing 2 µg of a “wrong” affinity purified antibody and 1 µg of BMAP-18 peptide. A positive control consisted of 50 µL of PBS containing 2 µg of anti-BMAP-18 affinity purified antibody with 1 µg BMAP-18 peptide. After 18 hours of incubation, 10 µL of a suspension of washed Dynabeads (M-280; sheep anti-rabbit IgG; cat no. 112.03D) were added to all of the experimental samples, the mixtures were incubated for 2 hours at room temperature and the beads from each were washed three times with PBS before eluting the peptides with 25 µL of 5% acetic acid. The 25 µL samples were Zip-Tipped (C18; P10 tip size; Cat No. ZTC18S960; Millipore Corporation, Billerica, MA) to concentrate the eluted peptides and to remove salts and then the peptides were eluted in 1 µL of 50% acetonitrile/0.1% trifluoroacetic acid. MALDI-TOF analysis was performed by spotting the eluted peptides (all of the 1 µL) onto the wells of a Voyager, 100 position, stainless steel MALDI plate (Applied Biosystems, Foster City, CA). After drying, the peptide spots were covered with 1 µL of matrix (0.5% alpha-cyano-4-hyrdoxycinnamic acid/0.18% ammonium citrate/70% acetonitrile/0.1% trifluoroacetic acid). An Applied Biosystems Voyager DE-STR mass spectrometer (Applied Biosystems, Foster City, CA) running in delayed extraction, reflectron mode was used to acquire MALDI-TOF data.

## Results

### The effect of BMAP-27 and BMAP-18 on kinetoplastid parasites and the bacterial symbiont of tsetse, *Sodalis glossinidius*

**Figure 2:**
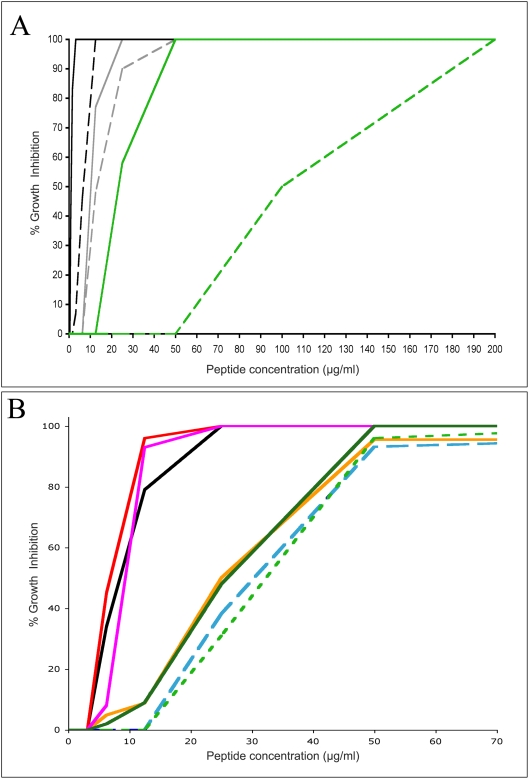
Inhibition of parasite growth by BMAP-27 and BMAP-18 peptides. MIC assays were performed on trypanosomes and *Leishmania* using alamarBlue as described in the Materials and Methods. Panel A: Comparison of full length and truncated host defense peptides. *T. b. brucei* 427.01 BSF (black lines), PCF (gray lines) and *Sodalis glossinidius* (green lines) were tested for sensitivity to BMAP-27 (solid lines) and BMAP-18 (dashed lines). Percent inhibition is shown as a function of peptide concentration. Panel B: Growth inhibition of a variety of kinetoplastid parasites by BMAP-18. *T. b. brucei* 427.01 PCF (black line), *T. b. brucei* TSW196 PCF (magenta line), *T. b. rhodesiense* ViTat 1.1 PCF (solid green line), *T. b. gambiense* U2 PCF (red line), *T. congolense* IL3000 PCF (orange line), *T. danilewskyi* TrCa (dashed green line), *L. donovani* LD3 (dashed blue line). The results shown are representative of more than 10 individual experiments (Panel A) or 4 individual experiments (Panel B) performed over a one year period.

To compare the growth inhibitory activity of the full-length bovine cathelicidin BMAP-27 with its truncated form, BMAP-18, both BSF and PCF of *T. b. brucei* parasites were cultured in the presence of varying concentrations of the peptides. After incubation for 66–72 hours, the metabolic activity of the organisms was determined using alamarBlue substrate. The results are shown in Figure 2A. Both life cycle stages of the trypanosomes were inhibited by BMAP-27 at extremely low concentrations (50% inhibition at <2 µg/mL for BSF, solid black line; and <10 µg/mL for PCF, solid gray line). The truncated BMAP-18 exhibited similar effects, with strong inhibition of both BSF and PCF trypanosomes observed at low doses of the peptide (50% inhibition at 8 and 12 µg/mL respectively). In comparison, a bacterial symbiont of tsetse, *Sodalis glossinidius* was not killed as effectively (green lines), especially with BMAP-18 (dashed green line) that required at least a ten-fold higher concentration (50% inhibition at 100 µg/mL) of peptide to effect similar levels of growth inhibition.

To test the parasite specificity of BMAP-18, several species of kinetoplastid parasites were tested in inhibition assays. The results are shown in Figure 2B. *T. b. brucei*, *T. b. gambiense* and *T. b. rhodesiense* PCF, members of the subgenus genus trypanozoon and species that are pathogens of domestic animals and humans, showed strong growth inhibition by low doses (50% inhibition at <10 µg/mL to a maximum of 25 µg/mL) of BMAP-18. *T. congolense*, a member of the subgenus Nannomonas, was also strongly inhibited by BMAP-18. Promastigote forms of *Leishmania donovani* parasites and the non-African trypanosome, *T. danilewskyi*, a pathogen of fish, were also highly sensitive to killing by BMAP-18.

### The effect of BMAP-27 and BMAP-18 on mammalian and insect cell lines

**Figure 3:**
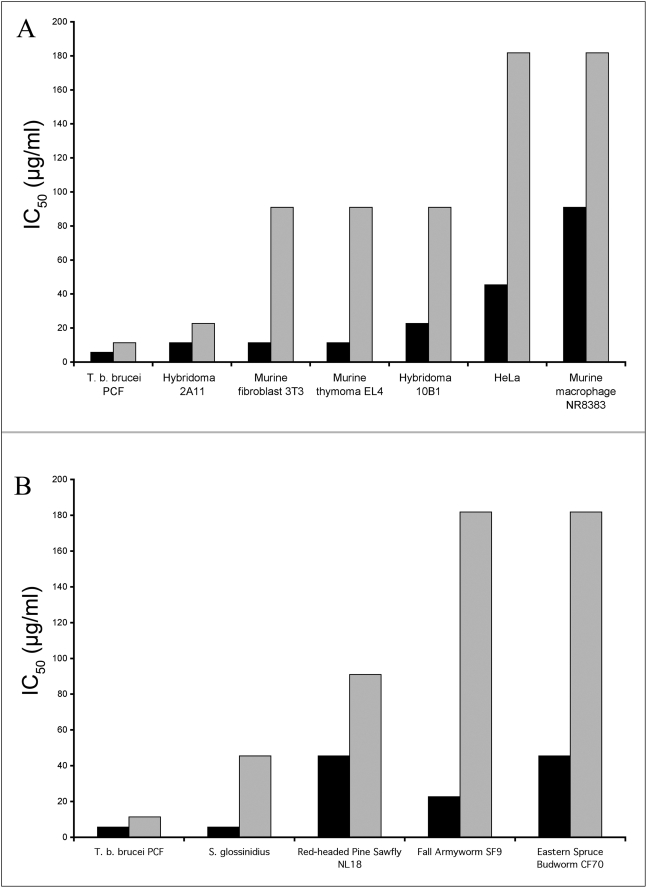
Comparative effects of BMAP-27 and BMAP-18 on growth of mammalian and insect cell lines. Inhibition assays using alamarBlue (as described in Materials and Methods) were performed on a variety of cell lines grown *in vitro*. A range of concentrations of peptides was tested against all cell lines. The concentrations of peptides required for 50% inhibition (MIC_50_) are shown. A representative parasite, *T. b. brucei* 427.01 PCF, treated under the same conditions, is included to indicate the marked difference in peptide concentrations required for inhibition of parasite and mammalian cell lines. Panel A: Mammalian cell lines. Panel B: Insect cell lines. Black bars: BMAP-27; Gray bars: BMAP-18. The results shown are representative of three individual experiments performed over a one year period.

Both mammalian cells and insect cells were tested in the alamarBlue assay with both forms of BMAP. The results are shown in Figure 3. All of the mammalian cell lines tested were less sensitive to BMAP-18 than to BMAP-27 (Panel A). Even the rapidly growing hybridoma cell line was less sensitive than *T. b. brucei* (shown on the left side of each of the panels) used as a comparative positive control. Similarly, BMAP-18 showed reduced cytotoxicity (approximately 2–8 fold) on all insect cell lines (Panel B) when compared with the parental BMAP-27 peptide.

### Physical effects of BMAP-18 on various kinetoplastid populations

**Figure 4:**
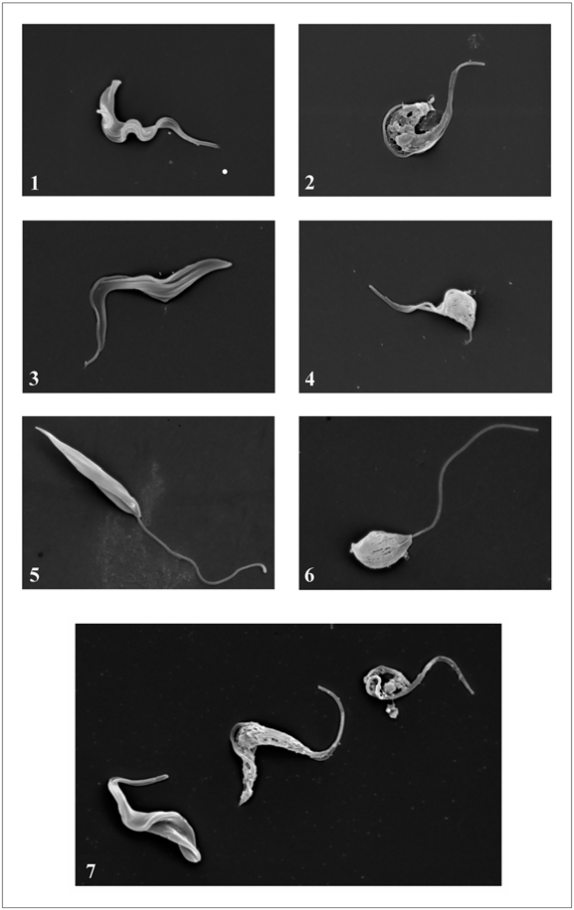
Scanning electron micrographs of trypanosomes and *Leishmania* before and after treatment with BMAP-18. Parasites in log-phase of growth were treated with BMAP-18 peptide (high concentration; 50 µg/mL) *in vitro* for 15 minutes before fixing and processing for microscopy. *T. b. brucei* 427.01 PCF, *T. b. rhodesiense* BSF and *L. donovani*. Panel 1: untreated *T. b. rhodesiense* BSF, Panel 2: BMAP-18-treated *T. b. rhodesiense* BSF. Panel 3: Untreated *T. b. brucei* 427.01 PCF, Panel 4: BMAP-18 treated *T. b. brucei* 427.01 PCF, Panel 5: untreated *L. donovani* LD3 promastigotes, Panel 6: BMAP-18 treated *L. donovani* LD3 promastigotes, Panel 7: BMAP-18 treated *T. b. brucei* 427.01 BSF trypanosomes seen in the same microscopic field. All parasites are shown at 5000× magnification (5 mm bar = 1 micron).

*T. b. brucei* 427.01 PCF, *T. b. rhodesiense* BSF and *L. donovani* populations were treated with high doses (50 µg/mL) of BMAP-18, fixed and examined by scanning electron microscopy at 5000× magnification (Figure 4). All three species of parasites showed membrane damage after treatment with BMAP-18 (Panels 2, 4, 6) whereas membranes of untreated parasites remained intact (Panels 1, 3, 5). Three individual *T. b. brucei* 427.01 PCF trypanosomes were photographed in the same microscopic frame in various stages of BMAP-18-based destruction after 15 min incubation (Panel 7). After 30 min incubation, no parasites had survived (not shown).

### Effects of BMAP-18 on rhodamine 123 retention in trypanosomes

**Figure 5:**
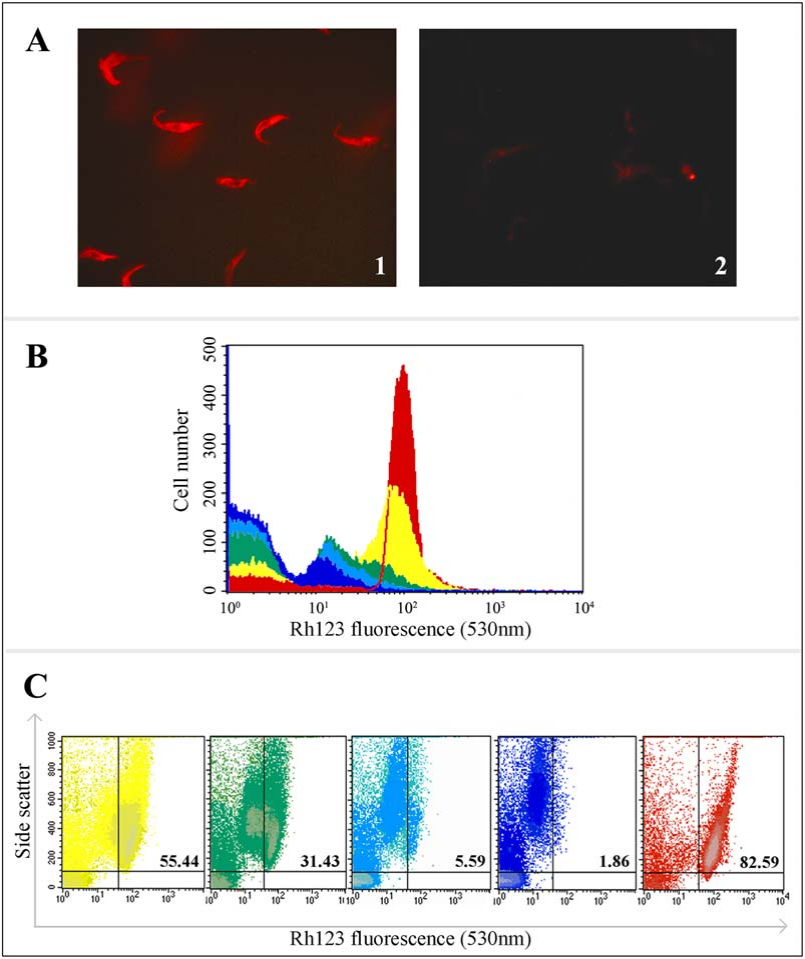
Analysis of trypanosomal mitochondrial activity by fluorescence microscopy and flow cytometry before and after treatment with BMAP-18. *T. b. brucei* 427.01 PCF were incubated with rhodamine123 as described in the Materials and Methods, examined by fluorescence microscopy and digitally photographed (Panel A1). The rhodamine 123 labeled trypanosomes were then treated with a low dose of BMAP-18 (5 µg/mL) and photographed again (Panel A2). Rhodamine fluorescence (FL-2) was also monitored by flow cytometry, before and after varying intervals of BMAP-18 treatment (Panel B). The different colours indicate different incubation times with the BMAP-18 peptide. Red: diluent control, 30 min; Yellow: BMAP-18 treated, 5 min; Green: BMAP-18 treated, 10 min; Light blue: BMAP-18 treated, 20 min; Dark blue: BMAP-18 treated, 30 min. The plot clearly shows decreasing rhodamine 123 fluorescence as the incubation period of BMAP-18 with the trypanosomes was extended from 0–30 minutes. A dot-plot comparison of this experiment (Panel C) showed the effects of BMAP-18 treatment on fluorescence intensity vs side scatter (cell granularity) over time. The numbers in the upper right quadrants of the dot-plots indicate the percentage of healthy cells with strong mitochondrial rhodamine fluorescence. In Panel B, the horizontal (X) axis indicates arbitrary fluorescence units and the vertical (Y) axis indicates the number of cells (events). In Panel C, the Y axis is SSC (side scatter).

Rhodamine123 is a cell permeant fluorescent dye that accumulates in mitochondria, which run the entire length of the trypanosome body. It is thought that an attraction of cationic rhodamine 123 molecules by the relatively high negative electric potential across the mitochondrial membrane may be the basis for the selective staining of mitochondria by rhodamine 123 in living cells. The dye at low concentrations exhibits no cytotoxicity [40]. It is used, among other things, to indicate mitochondrial membrane disruption. After incubating *T. b. brucei* 427.01 PCF with rhodamine123 and subsequently treating the same populations with low-doses (5 µg/mL) of BMAP-18, we assessed the dye retention of the cells by fluorescence microscopy (Figure 5). Trypanosomes were first treated with rhodamine 123 and photographed. These control (untreated) parasites showed bright red fluorescence distributed along the mitochondria (Panel A1). Rhodamine 123 labeled trypanosomes were then treated with BMAP-18 for 10 min and photographed again. This time no fluorescence was observed (Panel A2), indicating that the mitochondrial potential was disrupted, releasing the dye. These results were confirmed by flow cytometry over a 0–30 minute time course (Figure 5B). The plot clearly shows decreasing rhodamine 123 fluorescence as the incubation period of BMAP-18 with the trypanosomes was extended. A dot-plot comparison shows the effects of BMAP-18 treatment on mitochondrial fluorescence vs. side scatter (granularity) over time (Figure 5C). The diluent control (untreated trypanosomes after 30 min; far right panel) showed that approximately 83% of the parasites were alive and healthy as they had strong rhodamine 123-stained mitochondria and low granularity. After incubation with BMAP-18, the parasites remained intact and appeared to have a normal plasma membrane but showed decreasing rhodamine fluorescence and increasing granularity as the cells rounded up and underwent an apoptosis-like death. In other experiments (not shown) the vital dye fluorescein diacetate was retained by the parasites incubated with low levels (5 µg/mL) of BMAP-18, indicating that the integrity of the plasma membrane was maintained.

### Immunomodulatory activity of BMAP-27 and BMAP-18

**Figure 6:**
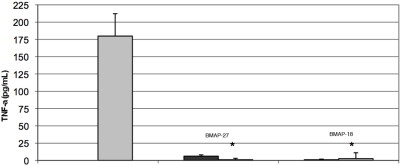
Inhibition of LPS-induced TNF-α secretion from human PBMC by BMAP-27 and BMAP-18. PBMC were incubated with 5 µg/mL of BMAP-27 or BMAP-18 for 30 minutes before treating with highly purified *Pseudomonas aeruginosa* lipopolysaccharide (10 ng/mL). After 4 hours, TNF-α was measured in the supernatants by ELISA. Gray bar: PBMC incubated with LPS alone. Black bars: PBMC incubated with BMAP-27 or with BMAP-18. The asterisks indicate the responses after treatment with LPS (p<0.01 comparing LPS alone to LPS+peptide). Three complete biological replicates were performed and the standard errors are shown.

Both BMAP-27 and BMAP-18 were tested for their ability to induce release of the cytokines MCP-1, Gro-α and TNF-α from human PBMC. In four separate experiments, BMAP-27, at a range of concentrations, stimulated release of MCP-1 and Gro-α cytokines, whereas BMAP-18 at the same range of concentrations did not. Neither BMAP-27 nor BMAP-18 directly stimulated the release of TNF-α from PBMC (data not shown). However, in three separate experiments, both BMAP-27 and BMAP-18, at physiologically relevant concentrations, strongly inhibited LPS-induced TNF-α secretion from PBMC (Figure 6).

### Immunodetection of BMAP-18 peptide in blood and plasma

A modified “peptide ELISA” (see Methods) was used to measure the titres of rabbit anti-BMAP-18 antibodies without interference from the linker moieties used to couple peptides to the KLH carrier used for immunization. Titres of rabbit antisera in excess of 1∶6400 were obtained. To test the utility of these antisera for detection of BMAP-18 in complex antigenic mixtures, a dilution series of human serum was made and 0.5 µg BMAP-18 peptide was spiked into 100 µL of each dilution for antigen coating of ELISA plates. The rabbit antibodies detected the BMAP-18 peptide even in undiluted human serum (data not shown) thus affinity-purifed anti-BMAP-18 antibodies were prepared for use in a novel immuno-mass spectrometry assay.

**Figure 7:**
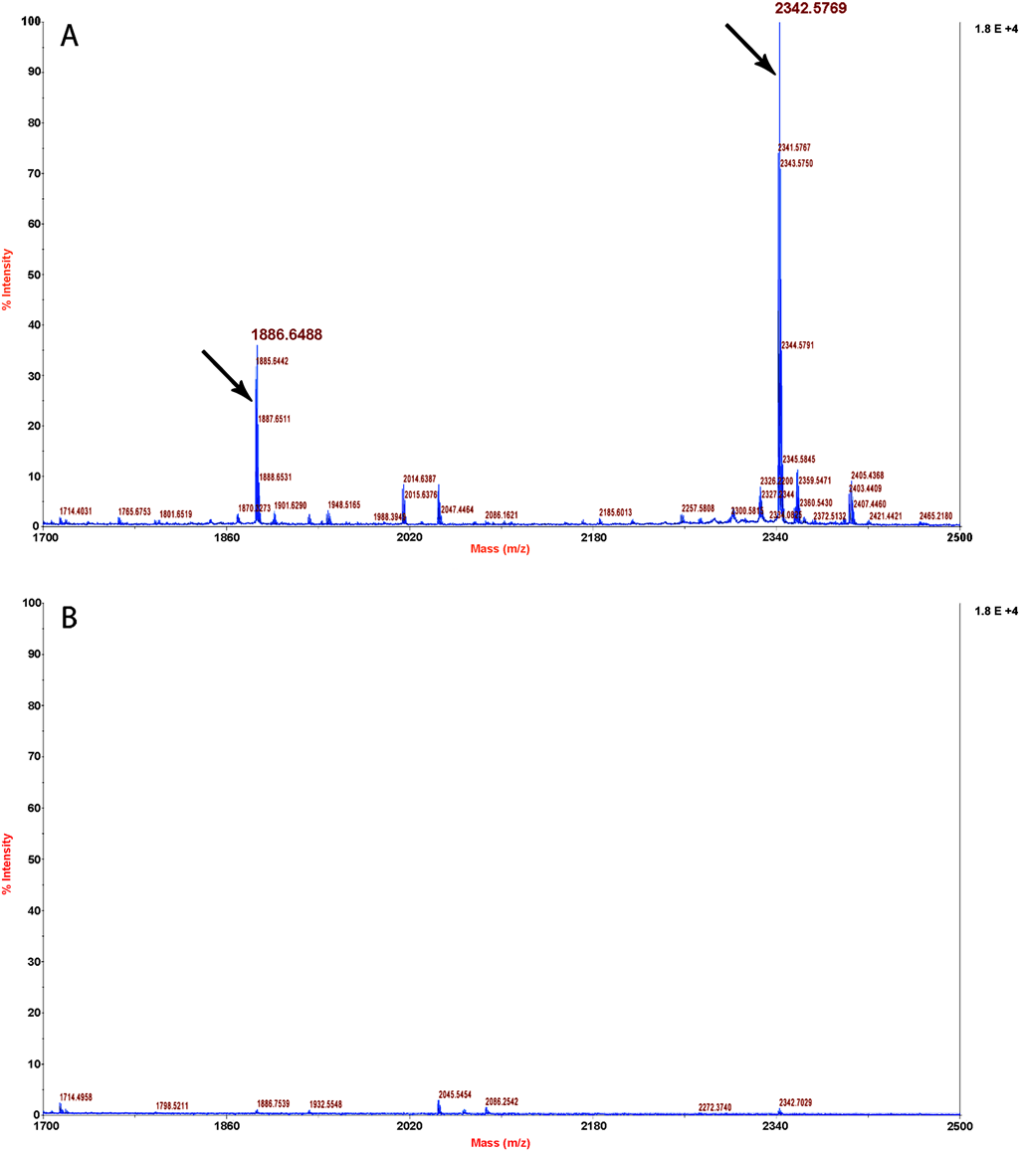
Mass spectrometric detection of intact BMAP-18 peptide after immunoenrichment from human plasma by anti-BMAP antibodies. MALDI-TOF mass spectrometry was used to determine the peptide mass spectra of eluates from Dynabeads used to capture antibody-BMAP-18 complexes from human plasma. Panel A shows the spectrum obtained from 50 µL of human plasma spiked with 1.0 µg of BMAP-18 peptide after capture of BMAP-18 with affinity-purified anti-BMAP-18 antibody. Panel B shows the spectrum obtained when no peptide was spiked into the plasma. This negative control reveals that no off-target binding occurred. In Panel A the average mass is the peak indicated by the arrow at 2342.57 m/z. A smaller peak at 1886.64 m/z was also seen and is likely a degradation product of BMAP-18. Both Panel A and Panel B show accumulated spectra. This experiment was repeated twice and similar results were observed.

Enrichment of BMAP-18 from spiked human whole blood, plasma or serum was attempted using affinity-purified anti-BMAP antibodies and capture of the antibody-peptide complexes by magnetic Dynabeads, followed by detection of the eluted peptide by MALDI-TOF mass spectrometry. The results are shown in Figure 7. We easily detected intact (non-degraded) BMAP-18 (2342.57 m/z; Panel A) after 18 hours of incubation in human blood or plasma, indicating that it is remarkably stable and suggesting that it would remain intact for a reasonable time in the blood of humans if it were used as a potential therapeutic. In contrast, we were unable to detect intact BMAP-18 in human serum (data not shown), perhaps because of the increased proteolytic activity in serum after activation of the clotting cascade.

## Discussion

Many of the parasites that cause disease remain problematic since in the absence of effective vaccines, control measures rely heavily on prophylactic or therapeutic drugs that have undesirable characteristics. Many therapeutics are prohibitively expensive to the people that need them and have unacceptable toxicity. In addition, there is increasing drug resistance in the target populations of parasites, for example in African trypanosomes [2] and *Leishmania* [3]. There is thus a need for new anti-parasite effectors that have more desirable features, such as a broad spectrum of activity, low toxicity for the recipient and a low propensity to induce microbial resistance. Host defense peptides have many of these features thus were chosen for our initial work on protozoan parasites [15] and for the work reported here.

HDP are a class of antimicrobial agents with promising applications across the spectrum of human and animal disease [4]. These peptides don't simply target a single species of microbial pathogen but work on two fronts. First, HDP can kill a broad range of microbial pathogens directly by perturbation of their plasma membranes or by permeating them and attacking an internal target. Second, HDP can selectively modulate innate host defenses [7]–[9]. Such an integrated mode of action is without question an attractive alternative to the standard use of antimicrobials, including the classic antibiotics. In fact, various HDP have shown potential as therapeutics for sepsis [42],[43] and bacterial [44] infections. Although these applications *in vivo* and other developments *in vitro* are encouraging, the practical use of these peptides *in vivo* still presents problems that must be overcome. Some HDP are inhibited by salt, even at physiological concentrations [45] and some peptides are toxic to mammalian cells [10]. In addition, some HDP (for example, the human cathelicidin LL-37) are too large for routine production by standard peptide synthesis techniques and some have proven difficult to express as recombinant peptides in bacteria or yeast due in part to toxicity issues. In the work presented here, we have addressed some of these issues by testing BMAP-18, a truncated form [10] of the bovine cathelicidin BMAP-27, in various assays *in vitro*, in order to assess the utility of this truncated peptide for potential application *in vivo*.

The highly trypanocidal BMAP-27 molecule [15] was first shown by Skerlavaj *et al* to be cytotoxic for mammalian cells, a problem partially solved by synthesizing a truncated form, BMAP-27 [1]–[18] (now called BMAP-18) that had the hydrophobic C-terminal segment removed [10]. In the work reported here, we compared BMAP-27 and BMAP-18 for their ability to kill a variety of species of protozoan parasites, including African trypanosomes, the infamous pathogens of humans and livestock in Africa, a trypanosome (*T. danilewskyi*) that is an economically important fish pathogen [24] and *Leishmania donovani*, an important human pathogen. Both forms of the peptide effectively inhibited all of these species *in vitro*, showing a broad range of specificity. Importantly, when compared to BMAP-27, BMAP-18 showed only a slight reduction in its ability to kill these parasites (approximately 2 fold more peptide required for 50% killing) and showed reduced toxicity (2–6 fold) on six different mammalian cell lines and three different insect cell lines. Thus, consistent with the findings of Skerlavaj *et al* [10], removal of the C-terminal 9 amino acids from BMAP-27 resulted in a molecule with greatly reduced cytotoxicity for non-microbial cells.

BMAP-18 killed both major life cycle stages of African trypanosomes (BSF and PCF) but was much less effective at killing the bacterial symbiont, *Sodalis glossinidius*. These results support the idea that BMAP-18 could potentially be used to treat trypanosome-infected animals or to inhibit the parasites in their tsetse vector. The latter could be attempted by interfering with trypanosome development in tsetse, perhaps by expression of the trypanocidal peptide in *Sodalis glossinidius* using paratransgenesis strategies [46]. BMAP-18 was also toxic to the fish pathogen *Trypanosoma danilewskyi* and to free-living *Leishmania donovani* promastigotes. We did not test whether or not BMAP-18 can penetrate *Leishmania* infected macrophages to kill the intracellular amastigote forms so its therapeutic potential for the Leishmaniases remains unknown.

BMAP-18 was shown to induce an apoptosis-like cell death in African trypanosomes and at higher concentrations caused membrane damage and subsequent death of the parasites by necrosis. We also tested whether or not BMAP-18 has immunomodulatory potential. Both the parent molecule, BMAP-27, and its truncated form, BMAP-18, failed to directly stimulate TNF-α secretion from HPBL but also strongly inhibited TNF-α release from LPS-stimulated HPBL. This may have practical implications for use of BMAP-18 as a therapeutic since TNF-α is a potent inflammatory cytokine that negatively affects trypanosome-infected animals. It will be of particular interest to test the bovine-derived BMAP-18 molecule for treatment of trypanosomiasis in cattle since there is evidence that a cathelicidin can be effective in treatment of disease in the species of animal from which the HDP was derived [47].

Since relatively large amounts of BMAP-18 will be required for testing in animals, it is of interest that stable integration of the BMAP-18 coding sequence and expression of BMAP-18 RNA has been achieved in transgenic potato plants (Teresa Francescutti, Biochemistry Dept, University of Victoria, personal communication). So far however, detection of the peptide itself has not been accomplished.

In anticipation of *in vivo* testing, we developed antibodies to BMAP-18 for measurement of the peptide in the blood of treated animals or in the midgut or hemolymph of tsetse. Even after 18 hours of incubation in freshly prepared, undiluted whole blood or plasma, intact (non-degraded) BMAP-18 was detected after antibody enrichment followed by MALDI-TOF mass spectrometry. This is encouraging because quantitative detection of BMAP-18 by standard immunological techniques, such as sandwich ELISA, will be problematic, since as a very small peptide, BMAP-18 likely does not contain two distinct epitopes, the minimal requirement for such assays. The fact that we could detect low levels of BMAP-18 by mass spectrometry, after enrichment of the peptide from human blood or plasma using a single antibody, bodes well for development of assays such as iMALDI [48] or SISCAPA [49] for quantitation of BMAP-18 and other HDP in complex biological fluids.

### Conclusion

Host defense peptides show great promise as therapeutics for a variety of infectious diseases. Here we have shown that BMAP-18 exhibits potent killing activity *in vitro* against a broad range of socioeconomically important parasites that infect humans, cattle and fish. However, for therapeutic applications *in vivo*, there are ongoing concerns about HDP toxicity, serum sensitivity and stability of the effector peptides in the blood of treated animals. In addition, there is evidence that resistance to direct anti-microbial killing by cationic peptides may arise, at least in some bacteria (50), thus any immunomodulatory effects of HDP are a welcome adjunct to direct killing. We have, in part, addressed these concerns for BMAP-18. First, BMAP-18 exhibited reduced toxicity on mammalian and insect cell lines. Second, BMAP-18 killing activity against several species of free-living parasites was potent in the presence of 10% fetal bovine serum in the culture medium used. Third, BMAP-18 could be detected by mass spectrometry in an unaltered form after enrichment from both whole blood and plasma using anti-peptide antibodies. Fourth, it was shown that BMAP-18 strongly inhibited LPS-induced release of TNF-α from leukocytes. Since TNF-alpha is one of the causes of cachexia (wasting) associated with African sleeping sickness (51) and is involved in immunosuppression in trypanosome infected animals (52), the data suggest that BMAP-18 is an excellent candidate for testing *in vivo* as a therapeutic that would directly kill trypanosomes while helping to maintain the overall health of the infected host.
